# Supplementary material for: Planktic foraminifera form their shells via metastable carbonate phases
Source: Nat Commun. 2017 Nov 2;8:1265. doi: 10.1038/s41467-017-00955-0 (PMC5668319; doi:10.1038/s41467-017-00955-0)
Supplement: Supplementary file 1 — Supplementary Information [file 41467_2017_955_MOESM1_ESM.pdf]

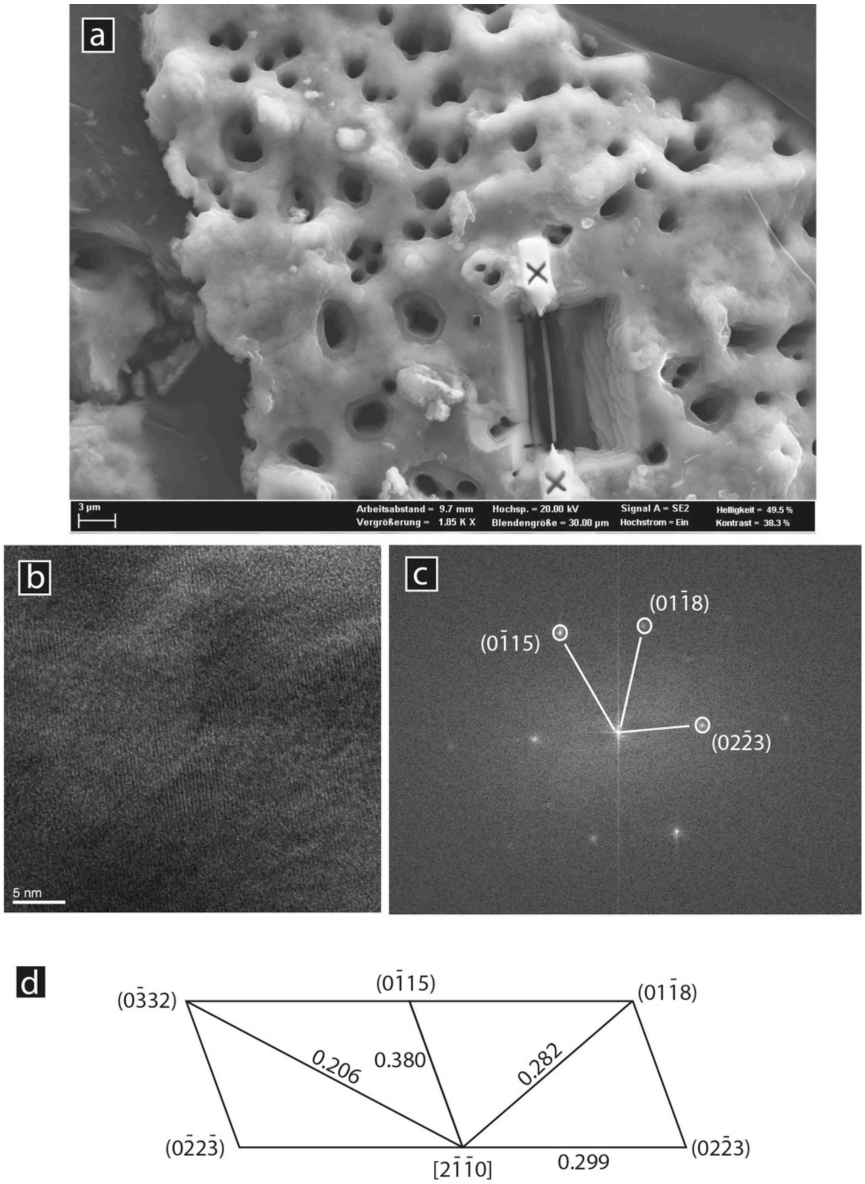

Angle between planes	calculated	observed
$01\bar{1}8/02\bar{2}3$	44.5°	42.76°
$01\bar{1}8/0\bar{1}15$	64.5°	66.54°
$00013/01\bar{1}8$	26.2°	27.15°
$00013/0\bar{1}15$	37.8°	39.38°
$0\bar{1}15/0\bar{2}2\bar{3}$	70.4°	70.70°

Supplementary Figure 1: Results from *Neogloboquadrina dutertrei.* (a) SEM image of a fragment of the final chamber of a *N. dutertrei* shell with FIB pit of foil #4186. (b) HR-TEM image of area shown after Fourier Transform analysis and indexed as vaterite twin cell A in (c). (d) Schematic indexed crystallographic cell shown in (b, c) with a list of the angles between crystallographic planes as observed, vs calculated angles using the hexagonal vaterite model proposed by *(1)*.

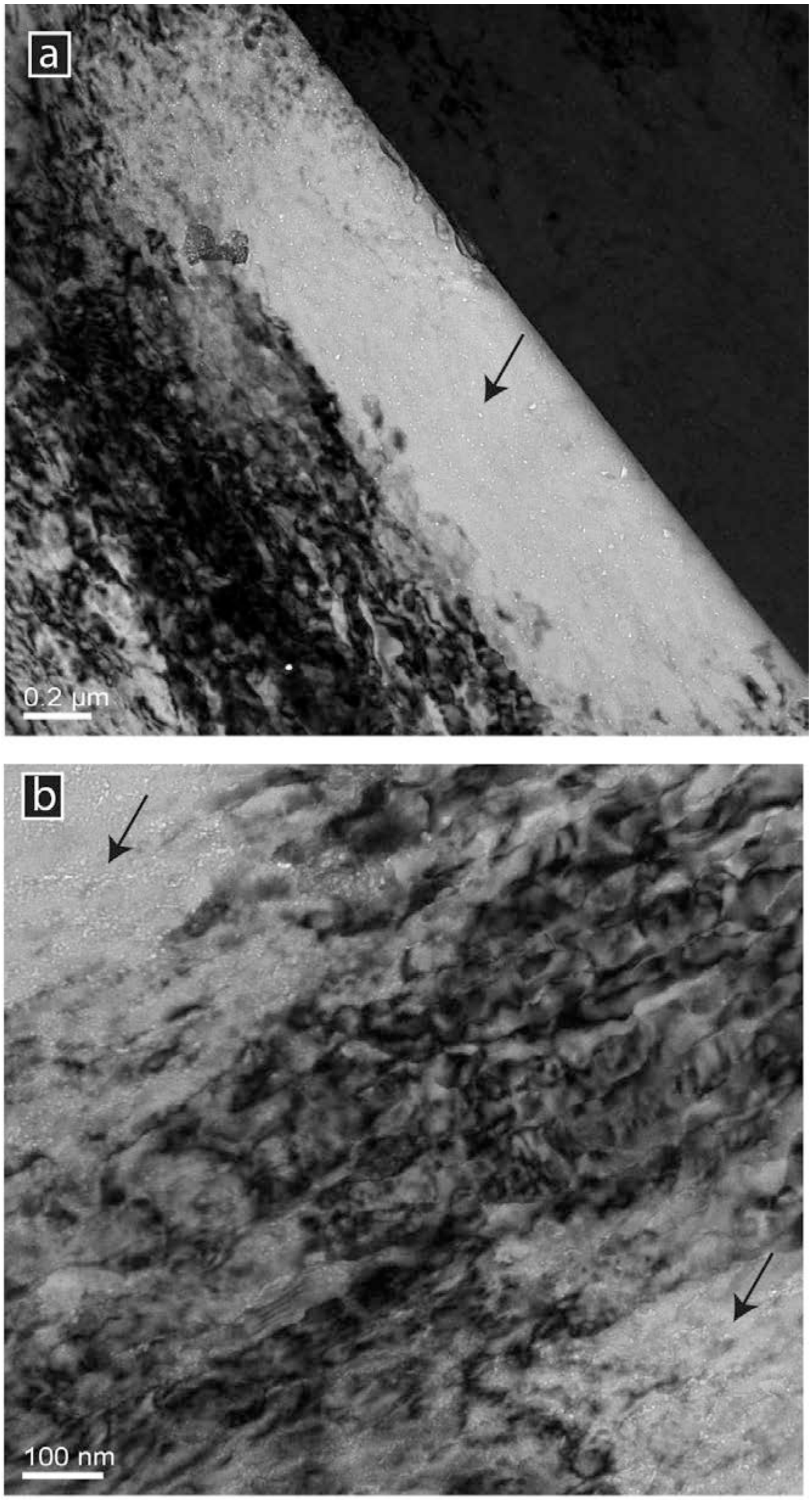

Supplementary Figure 2: Nanopores in *O. universa.* (a) Bright-field image showing the speckled contrast due to individual nanocrystals and scattered pores (arrow) in *O. universa* typical of biominerals. (b) typical nanostructure of irregular fibers with 'pores' (arrows).

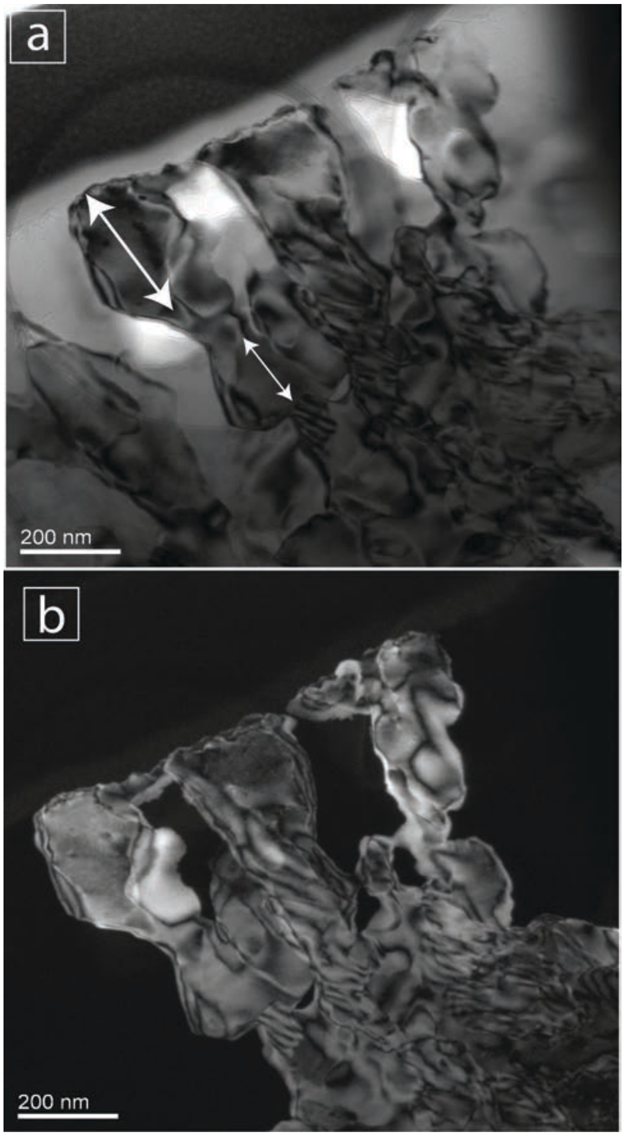

Supplementary Figure 3: Grain sizes in *N. dutertrei.* (a) Bright Field image of an area in foil #4186 (first analytical session) consisting of aggregated grains of vaterite. Approximate lengths of two grains are outlined by arrows. Grains are here defined by uniform diffraction contrasts. (b) Corresponding dark field image of the same area.

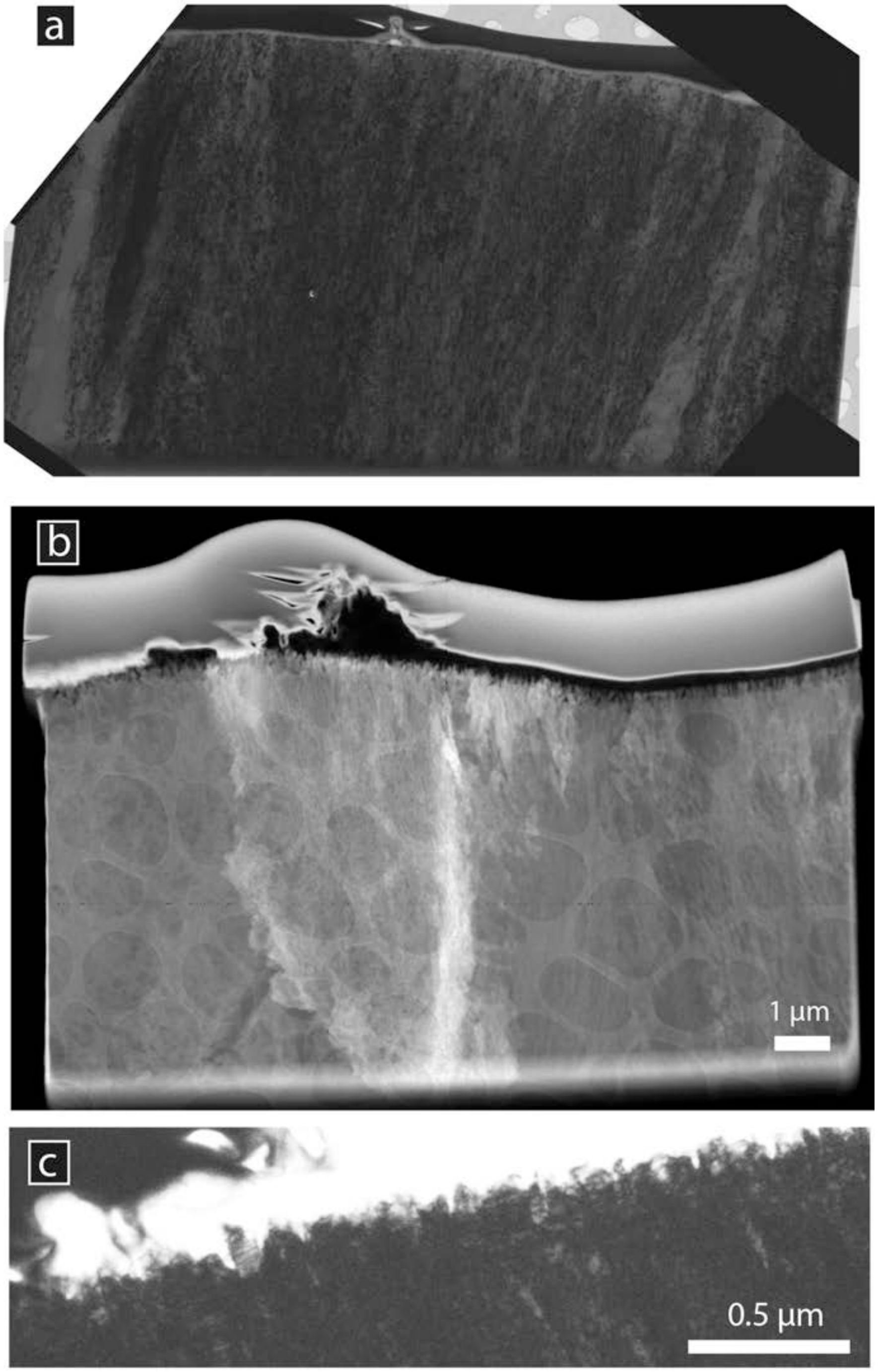

Supplementary Figure 4: Microstructure of *O. universa* shells. (a) Integrated image plate image of foil #4199 that shows an almost complete lack of diffraction contrast, indicative of a mesocrystal structure. (b) HAADF image of foil #4187 showing the ragged surface of the shell just below the protective Pt strip. (c) Bright-field image of the ragged surface area showing individual vaterite crystals.

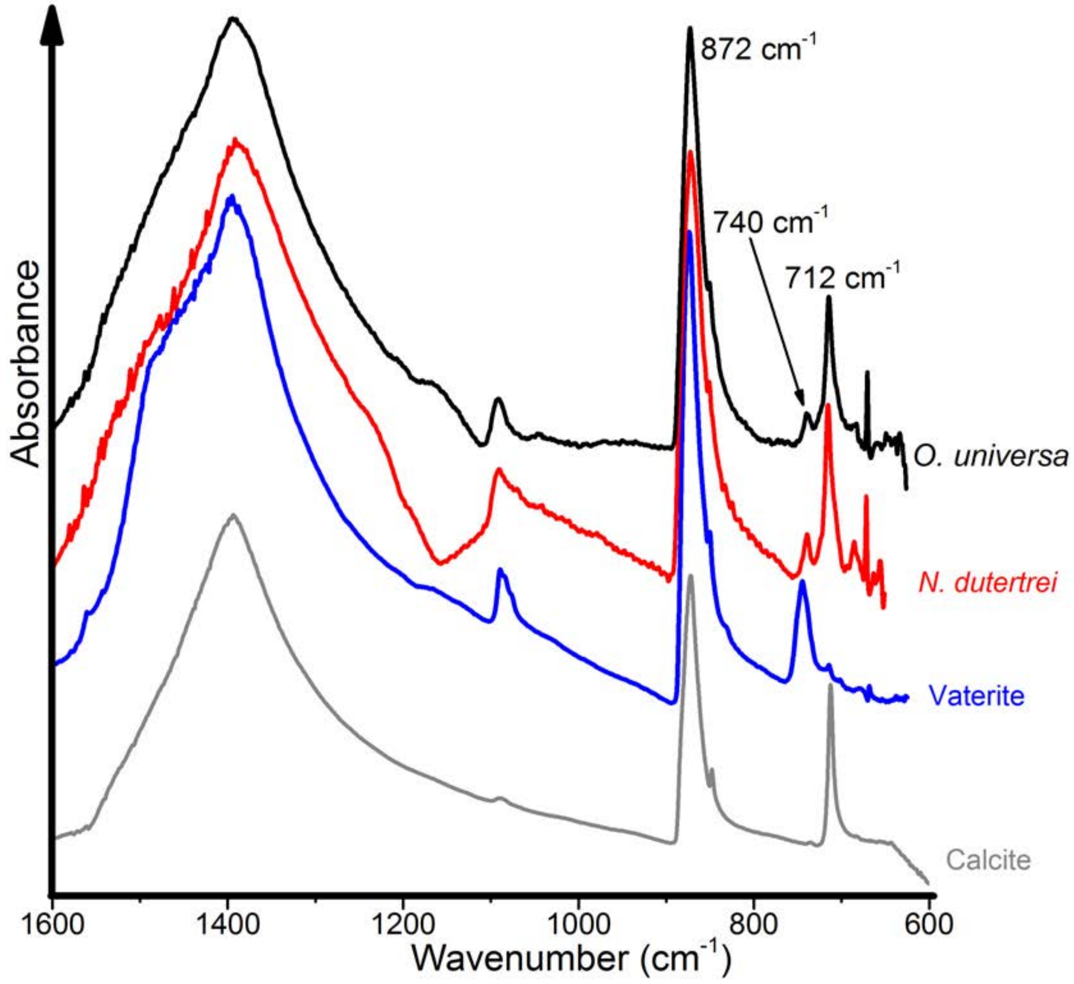

Supplementary Figure 5: Complete FTIR spectra (600-1600 cm^{-1}) for foraminifera shells. Spectra collected after dry storage, and compared to biogenic vaterite (*Herdmania momus* spicules) and geological calcite. The shells consist of a mixture of vaterite and calcite. The band at ca. 744 cm^{-1} is indicative for vaterite, while the band at 712 cm^{-1} is calcite *(2)*. The bands between 740 and 744 cm^{-1} are identical within the resolution of the spectra of 4 cm^{-1}. Bands for vaterite and calcite overlap at 872 cm^{-1} and at ca. 1400 cm^{-1}. Band assignments are given in Supplementary Table 2. All spectra are normalized to the band intensity at 872 cm^{-1}.

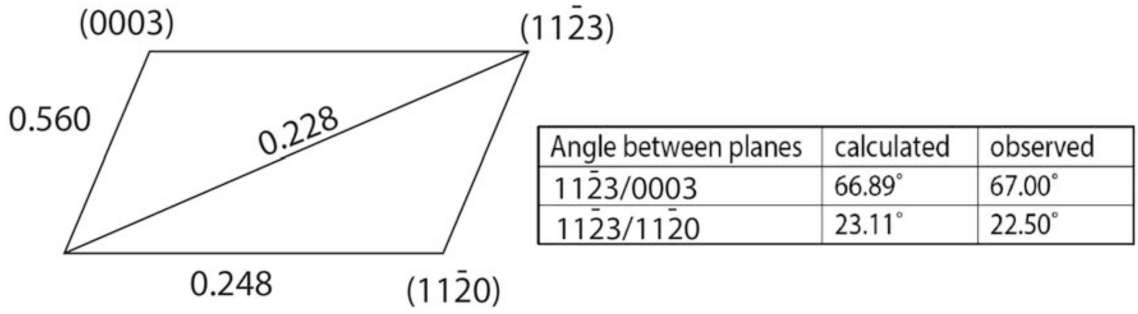

Angle between planes	calculated	observed
$11\bar{2}3/0003$	66.89°	67.00°
$11\bar{2}3/11\bar{2}0$	23.11°	22.50°

Supplementary Figure 6: Crystallographic analysis of HR-TEM images in Fig. 6. Lattice distances and measured angles are consistent with calcite and not with vaterite.

Supplementary Table 1. Measured d-spacings in *Orbulina universa* and *Neogloboquadrina dutertrei* shells compared with literature data. Several crystals from each foil were indexed by SAED or HRTEM and are compiled in the table.

**Observed**	**Vaterite ($P3_21$)**		**Calcite (R-3c)**	
d_{hkl} (nm)	***d_{hkl} (nm)***	***hkl***	***d_{hkl} (nm)***	***hkl***
0.841	0.844	0003	-	-
0.563	0.554	01-12	-	-
0.384	0.391	01-15	0.3855	10-12
0.307	0.306	02-23	0.303	01-14
0.280	0.282	01-18	-	-
0.299	0.290	02-23	-	-
0.245	0.248	20-26	0.249	11-20
0.227	0.229	12-32	0.2284	11-23
	0.225	12-33		
0.207	0.206	11-210	0.2094	20-22
0.192	0.1948	00013	0.1927	20-24
	0.190	30-35	0.9124	01-18
0.157	0.158	00016	0.1587	10-110

Supplementary Table 2: Band assignment for measured FTIR spectra from *Orbulina universa, Neogloboquadrina dutertrei* shells and *Herdmania momus* spicules. Spectral resolution is 4 cm^{-1}.

O. universa **(cm^{-1})**	***N. dutertrei*** **(cm^{-1})**	***H. momus*** **(cm^{-1})**	**Vaterite* (cm^{-1})**	**Calcite* (cm^{-1})**	**Bond Vibration**
872	872	872	877	876	carbonate ion out-of-plane bending mode (ν2)
740	740	743	744	-	carbonate ion in-plane bending mode (ν4)
712	712	-	-	712	carbonate ion in-plane bending mode (ν4)

*Band assignments for vaterite and calcite from *(2)*.

Supplementary References

1. Demichelis, R., Raiteri, P., Gale, J. D. & Dovesi, R. A new structural model for disorder in vaterite from first-principles calculations*. Cryst. Eng. Commun.* **14**, 44 (2012).

2. Andersen, F. A. & Brecevic, L. Infrared spectra of amorphous and crystalline calcium carbonate. *Acta Chem. Scand.* **45**, 1018–1024 (1991).
